# Porcine Bone Extracellular Matrix Hydrogel as a Promising Graft for Bone Regeneration

**DOI:** 10.3390/gels11030173

**Published:** 2025-02-27

**Authors:** Rotem Hayam, Shani Hamias, Michal Skitel Moshe, Tzila Davidov, Feng-Chun Yen, Limor Baruch, Marcelle Machluf

**Affiliations:** 1 Faculty of Biotechnology & Food Engineering, Technion—Israel Institute of Technology (IIT), Haifa 3200003, Israel; rotemh27@gmail.com (R.H.); shanihm17@gmail.com (S.H.); tzila937@gmail.com (T.D.); frankyyen86@gmail.com (F.-C.Y.); baruchl@bfe.technion.ac.il (L.B.); 2 The Interdisciplinary Program for Biotechnology, Technion—Israel Institute of Technology, Haifa 3200003, Israel; michalskitel@gmail.com

**Keywords:** extracellular matrix, bone graft, tissue engineering, bone defect, hydrogel

## Abstract

Bone defects resulting from trauma, tumors, or congenital conditions pose significant challenges for natural healing and often require grafting solutions. While autografts remain the gold standard, their limitations, such as restricted availability and donor site complications, underscore the need for alternative approaches. The present research investigates the potential of porcine-derived bone extracellular matrix (pbECM) hydrogel as a highly promising bioactive scaffold for bone regeneration, comparing it to the human-derived bECM (hbECM). Porcine and human cancellous bones were decellularized and characterized in terms of their composition and structure. Further, the ECMs were processed into hydrogels, and their rheological properties and cytocompatibility were studied in vitro while their biocompatibility was studied in vivo using a mouse model. The potential of the pbECM hydrogel as a bone graft was evaluated in vivo using a rat femoral defect model. Our results demonstrated the excellent preservation of essential ECM components in both the pbECM and hbECM with more than 90% collagen out of all proteins. Rheological analyses revealed the superior mechanical properties of the pbECM hydrogel compared to the hbECM, with an approximately 10-fold higher storage modulus and a significantly later deformation point. These stronger gel properties of the pbECM were attributed to the higher content of structural proteins and residual minerals. Both the pbECM and hbECM effectively supported mesenchymal stem cell adhesion, viability, and proliferation, achieving a 20-fold increase in cell number within 10 days and highlighting their strong bioactive potential. In vivo, pbECM hydrogels elicited a minimal immunogenic response. Most importantly, when implanted in a rat femoral defect model, pbECM hydrogel had significantly enhanced bone regeneration through graft integration, stem cell recruitment, and differentiation. New bone formation was observed at an average of 50% of the defect volume, outperforming the commercial demineralized bone matrix (DBM), in which the new bone filled only 35% of the defect volume. These results position pbECM hydrogel as a highly effective and biocompatible scaffold for bone tissue engineering, offering a promising alternative to traditional grafting methods and paving the way for future clinical applications in bone repair.

## 1. Introduction

Bone, the structural support of the body, also functions as a reservoir of minerals, protects vital internal organs, and contributes to the maintenance of the body’s acid–base equilibrium. As it is a dynamic tissue, the bone undergoes a continuous process of remodeling, adapting to the varying physiological demands [1,2]. In cases of trauma, most bone defects can self-heal, a process that prominently involves the migration of mesenchymal stem cells (MSCs), which differentiate into chondrocytes and osteoblasts followed by the subsequent formation of new bone tissue [3]. During regeneration, a wealth of chemical and mechanical signals are transmitted through the bone extracellular matrix (ECM), and ECM remodeling is coordinated until the full restoration of the bone is accomplished [4]. However, large-scale defects impediment the natural ability of the bone to heal and do not allow for complete fracture healing. These large bone defects mostly result from traumatic injuries, tumor resections, or congenital defects [5]. The regeneration of these large defects requires the utilization of bone grafts, with autografts currently considered the gold-standard treatment. Nevertheless, these autografts suffer from several problems such as limited availability and potential for complications at the donor site [6]. Allografts represent the second most common bone-grafting approach worldwide and are available in various forms, including demineralized bone matrices (DBMs) [2,7]. In contrast to autografts, allografts are associated with the risks of immunological reactions, the potential transmission of infections, and elevated failure rates in long-term usage. Accordingly, bone tissue engineering was suggested as a promising approach for bone regeneration, surpassing the constraints of traditional implants [8,9,10]. The inherent role of the ECM in providing mechanical scaffolding, comprising a sophisticated assembly of structural proteins, carbohydrates, and signaling molecules, extends beyond its role in tissue support [11]. The nanostructured ECM, particularly within bone tissues, reinforces collagen fibers with hydroxyapatite crystals, fostering a resilient and flexible framework that supports the adhesion, proliferation, and differentiation of various bone cells [10,12,13,14]. This versatile ECM functionality, demonstrated in vitro and in vivo [15,16], has motivated its application as a biomaterial in diverse biomedical contexts, where mechanical and biological support is required, particularly in bone regeneration [11,17]. In the context of xenotransplantation, where bone tissue is transplanted across species, the ECM’s pivotal role takes on added significance. Although non-human primates are phylogenetically closer than other species to humans, their candidacy for clinical xenotransplantation is hampered by ethical concerns, the substantial risk of cross-species infection transmission to humans, logistical challenges in breeding, and disparities in organ size [18]. Here, porcine origin emerges as a favored alternative, leveraging the significant molecular similarity between porcine and human tissues, due to the highly conserved ECM molecules across species [19]. Furthermore, the similarities in porcine organ size and physiology to humans, and the ability to breed pigs rapidly, render it a widely available and practical source option [20]. This positions the porcine-derived ECM as a practical and widely available source for bone grafts, bridging the gap between the intricate cellular scaffold function of the ECM and its application in addressing complex challenges associated with cross-species tissue transplantation. Previous studies have explored the use of porcine-derived ECM hydrogels [21] or scaffolds utilizing them [22] for bone regeneration, showing promising results in vivo. Nevertheless, tissue-specific bone ECM has been shown to promote increased osteogenic differentiation and bone regeneration through bone-specific proteins and minerals essential for osteogenesis [23,24]. The current study was therefore designed to investigate the feasibility of utilizing porcine-derived bone ECM (bECM) hydrogel as a highly available osteoinductive scaffold for the regeneration of bone tissue. To achieve this, we conduct a comparative analysis between the porcine bECM (pbECM) and human bECM (hbECM), followed by a direct evaluation of the pbECM hydrogel’s efficacy as a bone graft, thus elucidating its potential advantages and translational prospects.

## 2. Results and Discussion

### 2.1. Results

#### 2.1.1. Validation of Acellularity

**Figure 1:**
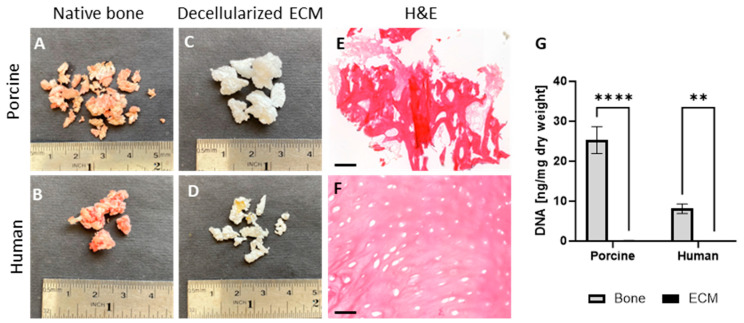
Decellularization process of bECM from cancellous bone tissue. Porcine (**A**) and human (**B**) bone fragments. Decellularized bECM from porcine (**C**) and human (**D**) bones. H&E staining of decellularized bECM from porcine (**E**) and human (**F**) bone fragments. (**G**) DNA content in porcine and human native bones and bECM, quantified using PicoGreen assay. ** *p* < 0.0011, **** *p* < 0.0001. Scale bars, 200 µm.

Our developed protocol for porcine cancellous bone tissue decellularization—using chemical, biological, and physical means—was addressed in terms of the acellularity of the resulting bECM. As can be seen from Figure 1, the decellularization process successfully resulted in cell-free bECM powder when using both human and porcine tissue sources. In addition, quantitative PicoGreen analysis for residual DNA content in the decellularized material showed that the DNA content was significantly lower compared to the native tissues in both human and porcine bECM (Figure 1G).

#### 2.1.2. bECM Protein Composition

**Figure 2:**
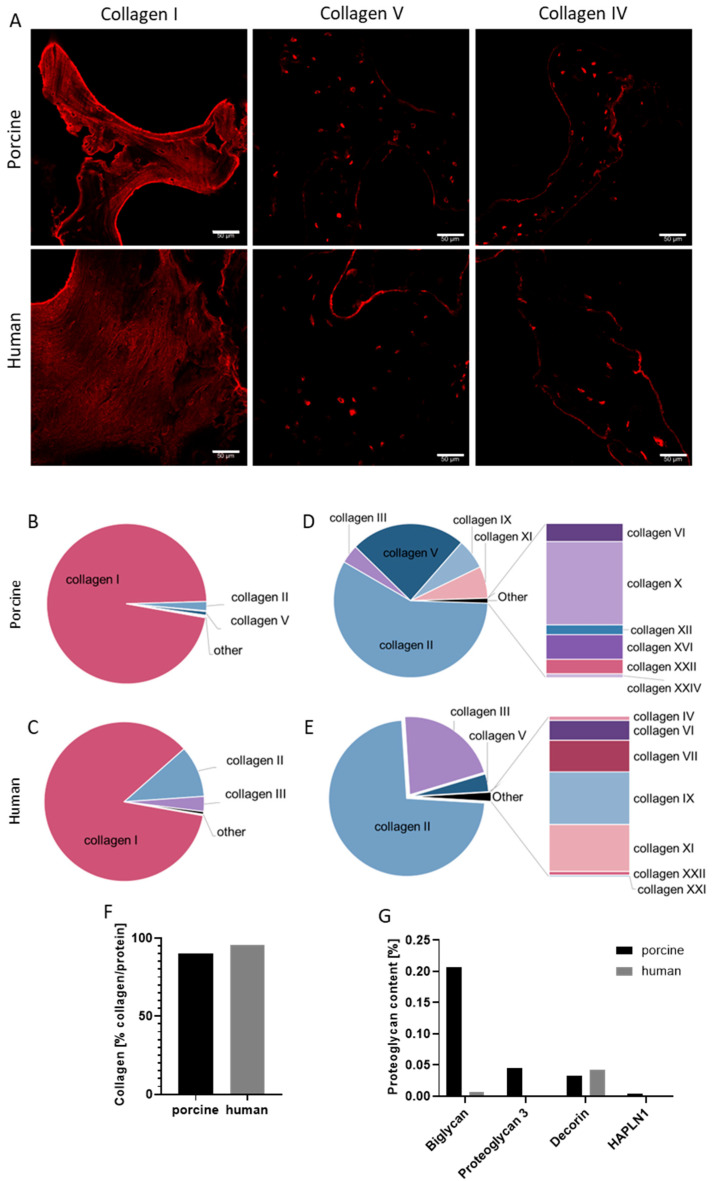
Protein composition of porcine and human bECMs. (**A**) Immunostaining of porcine and human bECMs for collagen I, collagen V, and collagen IV. Scale bars, 50 μm. (**B**,**C**) Content of the different collagen types in pbECM (**B**) and hbECM (**C**). (**D**,**E**) A more detailed analysis of the less abundant collagen types (excluding collagen type I) in pbECM (**D**) and hbECM (**E**). (**F**) Collagen percentage out of the protein content in porcine and human bECMs. (**G**) Proteoglycans content in porcine and human bECMs.

To characterize the resulting bECM, the preservation of ECM proteins was primarily examined by focusing on collagen type I, which usually constitutes 90% of the total collagen in bone tissue. Immunostaining of the bECM for collagen type I revealed a dense network in both porcine and human bECM samples. Collagen V, which regulates collagen type I’s fiber diameter and fibrillogenesis, was also present, albeit in smaller amounts than collagen I, with a similar distribution observed in both the porcine and human bECMs (Figure 2A). Collagen IV, known to reside in the ECM of different tissues, was also present in both bECM types. To comprehensively address ECM proteins, we further performed a semi-quantitative proteomic analysis, demonstrating the predominance of collagens in both bECM types. The proteomic analysis highlighted the higher number of proteins identified in the porcine bECM compared to the human bECM, with 126 and 75 proteins, respectively. Although both bECMs had a similar number of collagens—12 for the porcine bECM and 11 for the human bECM—not all collagen types were shared between them (Figure 2B–E). Collagen types I and II were the most abundant in both samples, with 87% and 2% in the porcine bECM and 82% and 10% in the human bECM, respectively. Significant levels of collagen type III were found in the human bECM at 3% compared to porcine bECM with only 0.1%. Similar levels of collagen type V were found in both bECM (0.7% in porcine and 0.5% in human); however, unique profiles emerged for less abundant collagens in each bECM (Figure 2D,E). Notably, collagen IV was not detected in the pbECM analysis but was observed in immunostaining (Figure 2A). Altogether, collagen constituted 95% of all proteins in the hbECM, while in the pbECM, it accounted for 90% (Figure 2F). Other protein types varied between the two bECMs (Table 1, Figure 2G). For example, three types of glycoproteins and two types of Small Integrin-Binding Ligand N-Linked Glycoproteins (SIBLINGs) were identified in the pbECM, while no proteins from these families were found in the hbECM. In addition, four types of proteoglycans were detected in the pbECM, while only two were found in the hbECM. Furthermore, biglycan, known for promoting collagen fibrillogenesis [25], was more abundant in the pbECM than in the hbECM (Figure 2G).

#### 2.1.3. Characterization of bECM from Porcine and Human

**Figure 3:**
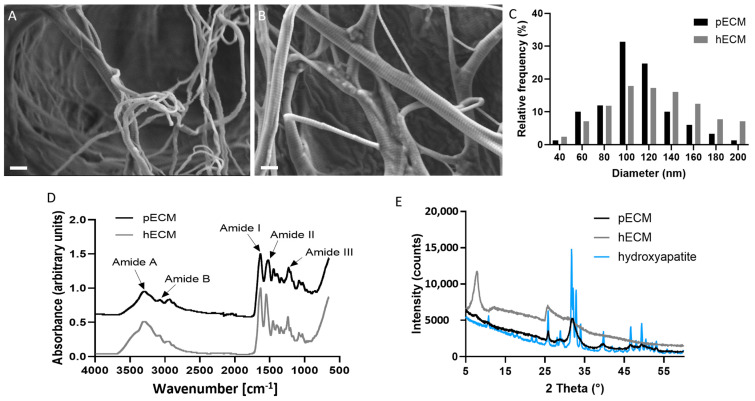
Characterization of bECM from human and porcine bone tissue. SEM images of (**A**) pbECM and (**B**) hbECM, scale bars 400 nm. (**C**) Fiber diameter distribution in pbECM and hbECM, analyzed from SEM images using ImageJ 1.53r. (**D**) Fourier transform infrared spectroscopy (FTIR) spectra of the pbECM compared to hbECM. (**E**) X-ray diffraction (XRD) analysis of the human and porcine bECM compared to commercial hydroxyapatite.

To assess the three-dimensional microstructure of the bECM, scanning electron microscopy (SEM) analyses were performed, demonstrating that both the porcine and human bECMs preserved the fibrous structure of the native collagen, characterized by a similar fiber diameter distribution with an average of 108 nm for the pbECM and 124 nm for the hbECM (Figure 3A–C). Fourier transform infrared spectroscopy (FTIR) was utilized to analyze alterations in the protein molecular structure of both human and porcine bECM, exhibiting similarity between the spectra of both bECMs (Figure 3D). Both the porcine and human bECMs presented similar bands for Amide A and Amide B. Amide A band (3280 cm^−1^) indicates the N–H stretching vibration, while the Amide B band (3100 cm^−1^) is associated with the asymmetrical stretching of C-H. Similar wavelength peaks were also demonstrated for Amides I, II, and III which are the main bands of collagen. Amide I (1633 cm^−1^) correlates to the carbonyl that is related to the stabilization of the triple helix structure, Amide II (1540 cm^−1^) represents the N-H bending, and Amide III (1230 cm^−1^) indicates the C-N stretching. Furthermore, the ratio between the intensity of the Amide III peak and the 1450 cm^−1^ peak was approximately ~1 for both bECMs, thus indicating the preservation of triple helix structures [26,27]. To assess the preservation of the crystalline structure of the minerals, the XRD spectra of the bECMs were compared to commercial hydroxyapatite. The porcine bECM displayed a diffraction peak at about 25.8° and a peak with a higher intensity centered around 32°, which indicates a significant content of inorganic components corresponding to HA. The human bECM, on the other hand, displayed a broad peak in the range of 25–32°, indicating a relatively low degree of crystallinity [28]. In addition, the hbECM showed a major peak at about 7.85°, and the pbECM showed a small peak at 7.5°, which corresponds to the periodicities of 1.1 nm characteristic of collagen’s molecular structure [29]. The swelling behavior was similar for the porcine and human bECMs (Figure S1), and their thermal decomposition was typical for the porcine ECM (Figure S2) [13].

#### 2.1.4. bECM-Derived Hydrogels

**Figure 4:**
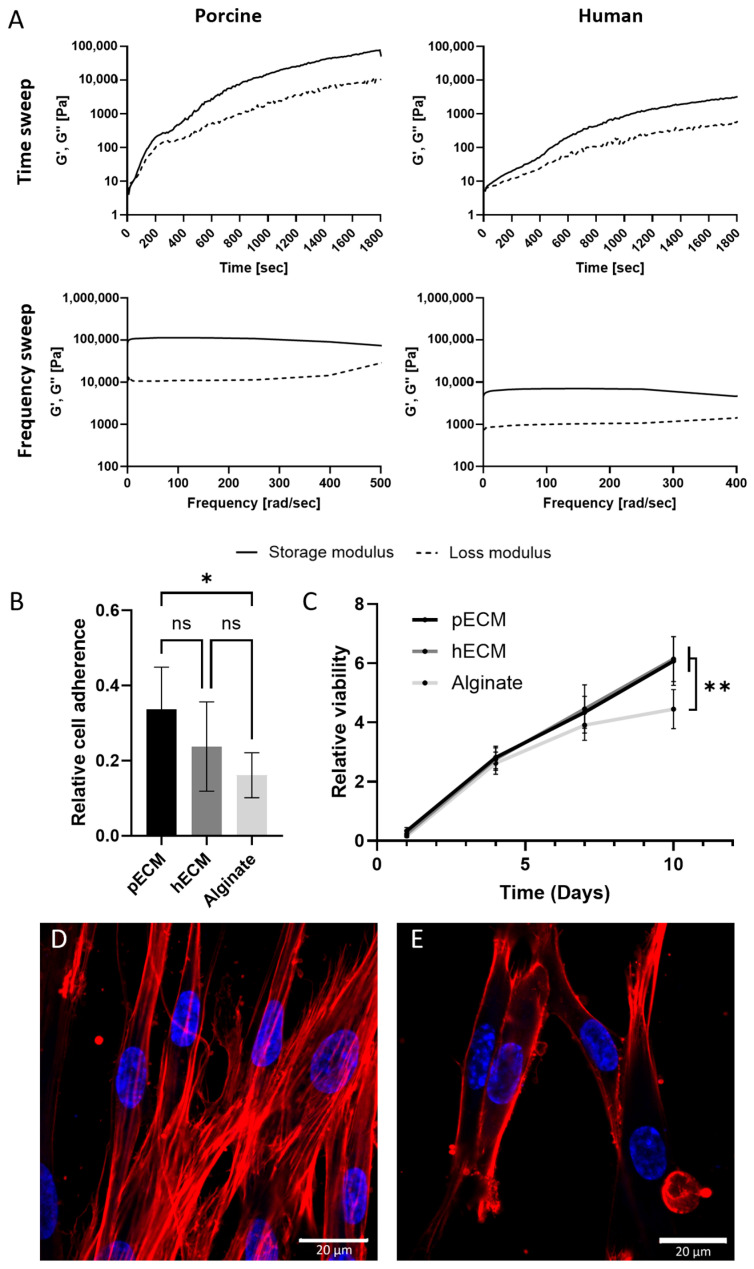
bECM hydrogels’ characterization. (**A**) Mechanical properties of the bECM hydrogels. (**Upper panel**): Time sweep rheological characterization, changes in G′ and G″ over time. (**Lower panel**): Frequency sweep rheological characterization, changes in G′ and G″ in ascending frequencies. Cytocompatibility of bECM hydrogels: (**B**) adherence of MSCs seeded to bECM hydrogels, and (**C**) viability over 10 days of culture, relative to the cell viability on a culture plate at day 1. * *p* < 0.05, ** *p* < 0.01. Confocal microscope images of MSCs on the (**D**) porcine and (**E**) human bECM hydrogels 10 days post-seeding. Red: Phalloidin (Actin), blue: DAPI (DNA). Scale bars 20 µm.

The porcine and human bECMs were processed into thermally induced hydrogels using enzymatic digestion, and the rheological properties of the bECM hydrogels were assessed through time and frequency sweep analyses (Figure 4A). The time sweep analyses were conducted after increasing the temperature to 37 °C to induce gelation, showing that both the storage modulus (G′) and loss modulus (G″) of the two hydrogels increased over time. Gelation started after approximately 30 s when the crossover point of the G′ and G″ moduli was observed [30]. In the frequency sweep analysis, hydrogels were subjected to small deformation oscillations across various frequencies to evaluate their response to deformations of different timescales. Throughout the frequency range, the storage modulus exceeded the loss modulus, and both moduli remained constant, indicating frequency independence, consistent with gel-like materials [31,32,33]. While increasing the deformation, the hydrogels experienced a progressing breakdown of their three-dimensional network. The pbECM hydrogel exhibited greater strength than the hbECM hydrogel, as evidenced by its higher G′ and its later deformation point at 380 rad s^−1^, compared to the hbECM hydrogel that showed a deformation point at approximately 250 rad s^−1^, as illustrated in Figure 4A.

After bone injuries, human mesenchymal stem cells (hMSCs) were recruited and differentiated into osteoblasts, the bone-forming cells, as a critical part of the regeneration process. Therefore, hMSC’s interactions with the bECM hydrogels can indicate the hydrogels’ potential as a graft for bone tissue engineering, thus supporting hMSC’s proliferation and promoting cell recruitment and differentiation. To investigate the interactions between MSCs and the hydrogels derived from human or porcine bECM, we evaluated the adhesion, viability, and morphology of MSCs cultured on these hydrogels. We used alginate as a control for these experiments, which is a common support material in tissue engineering scaffolds [34]. As can be seen from the relative viability identified on the first day, both hydrogels supported similar levels of MSC adherence (Figure 4B). The viability of the cells increased during the 10 days of culture, indicating that the cells had proliferated (Figure 4C). Higher proliferation rates were observed in both bECM groups compared to alginate, reaching approximately 20 times their initial numbers. Following 10 days of culture, the MSCs on the hydrogels were stained for actin fibers and nuclei, revealing a typical elongated morphology of the cells cultured on both the porcine and human bECM hydrogels (Figure 4D).

#### 2.1.5. Biocompatibility of the bECM Hydrogels

**Figure 5:**
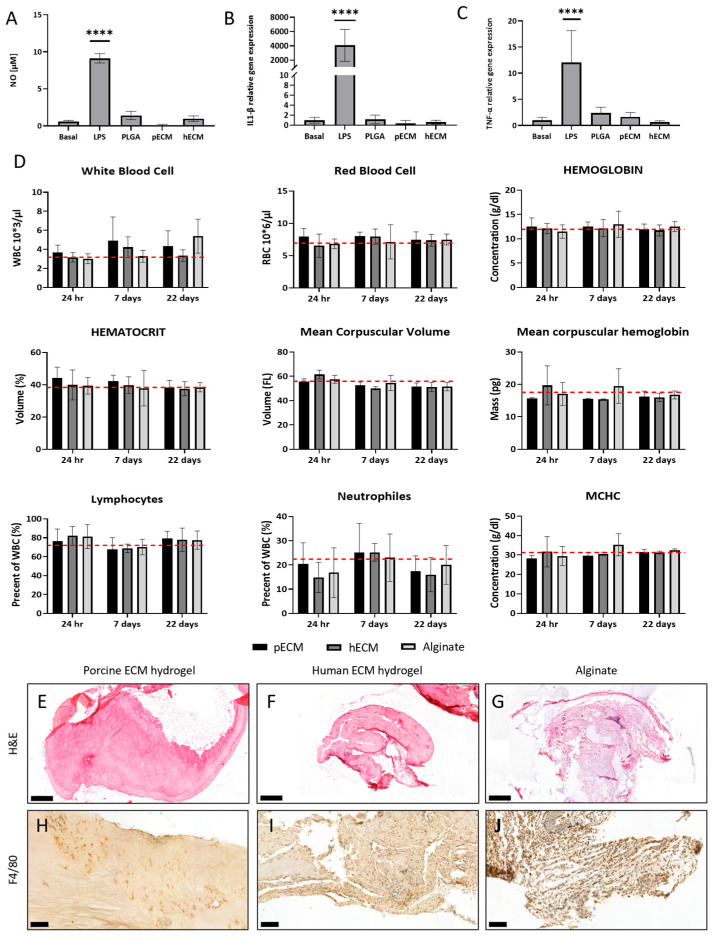
Biocompatibility of bECM. RAW macrophage stimulation assay in vitro: (**A**) NO secretion level, (**B**,**C**) expression of IL-1β (**B**) and TNF-α (**C**) mRNA, normalized to GAPDH. **** *p* < 0.0001. In vivo biocompatibility study: (**D**) complete blood counts of mice following subcutaneous implantation of bECM hydrogels. Number of white blood cells (WBCs) and red blood cells (RBCs), hematocrit volume, hemoglobin concentration, mean corpuscular volume (MCV), mean corpuscular hemoglobin (MCH), mean corpuscular hemoglobin concentration (MCHC), number of neutrophils, and lymphocytes, all plotted over three weeks following implantation. Dashed lines represent basal blood values for C57 black mice. (**E**–**G**) H&E staining (scale bars 500 μm) and (**H**–**J**) immunohistochemistry analyses for F4/80 (scale bars 100 μm) of hydrogels retrieved after 22 days post-implantation.

Implantation of a graft holds the risk of inducing a host reaction, which plays a critical role in determining the implant’s efficient integration and biological functionality. Therefore, the immunogenic and proinflammatory potential of the bECMs was assessed in vitro and in vivo. In vitro, we exposed the RAW macrophage cell line to the bECM from porcine and human bone tissues. Cells exposed to LPS served as the positive control, while untreated cells and cells exposed to PLGA served as the negative controls. A significantly lower NO secretion was obtained in all treatment groups compared to LPS (*p* < 0.0001), as can be seen in Figure 5A. Similarly, the expression levels of the proinflammatory cytokines interleukin 1-β (IL-1β) and tumor necrosis factor alpha (TNF-α) were significantly lower in all treatment groups compared to LPS (*p* < 0.0001), indicating the stimulation of LPS-treated macrophages but not of the bECM-treated groups (Figure 5B,C).

In vivo, the biocompatibility of the bECM hydrogels was assessed through subcutaneous implantation in C57 black mice. The hydrogels were compared to the non-immunogenic alginate (Figure 5D–J). The mice were sacrificed one, seven, and twenty-two days after implantation. Complete blood counts (CBCs) did not exhibit significant increases in the levels of white blood cells (WBCs), red blood cells (RBCs), hematocrit, hemoglobin, mean corpuscular volume (MCV), mean corpuscular hemoglobin (MCH), MCH concentration (MCHC), neutrophils, and lymphocytes in all treatment groups and at all time points (Figure 5D). Additionally, an analysis of the proinflammatory cytokine levels in the serum was conducted to further evaluate the immune response elicited by the hydrogels (Table 1). At 24 h post-implantation, the levels of IL1-β and IL-6 were significantly higher in the alginate group compared to the hbECM hydrogel group. By day 7, only IL-6 exhibited a significant decrease in the alginate group compared to the pbECM hydrogel group. After 22 days, both TNF-α and IL1-β showed a significant difference between the porcine and human bECM hydrogel groups. The proinflammatory cytokine IFN-γ levels revealed no significant difference between all groups at all time points, suggesting that the hydrogels did not induce a robust IFN-γ-mediated immune response. Histological assessment revealed cell migration towards all implants (Figure 5E–G). Nevertheless, in the pbECM hydrogel group, most cells accumulated at the hydrogel’s margins and significantly less infiltrating cells were observed compared to the hbECM hydrogel and alginate groups. Immunostaining for the macrophage marker F4/80 revealed a considerable macrophage presence among the migrated cells in the hbECM hydrogel group, suggesting an active immune response. In contrast, the pbECM hydrogel exhibited only a minor macrophage presence, indicating a potentially lower immunogenic response. In the alginate group, the majority of cells were stained positive for the F4/80 marker, reflecting a strong macrophage presence and highlighting a more pronounced immune reaction compared to the pbECM hydrogel group (Figure 5H–J).

Since no substantial immunogenic reaction was observed towards the pbECM hydrogel group, we chose to further evaluate it as a pro-regenerative bone graft in a bone defect model.

#### 2.1.6. Efficacy of the pbECM Hydrogel as a Bone Graft In Vivo

**Figure 6:**
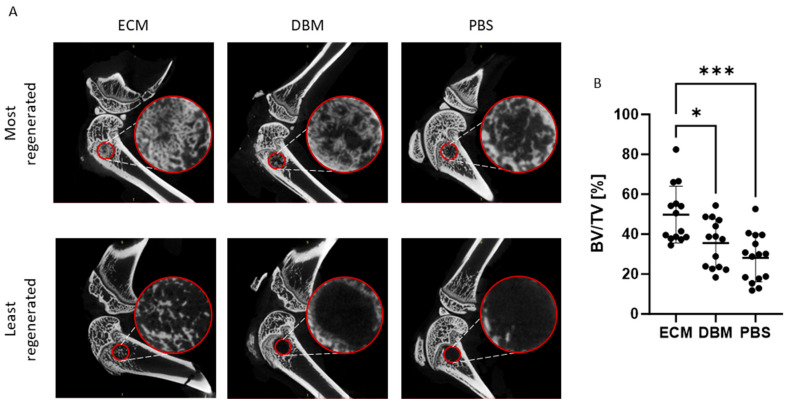
Efficacy of the pbECM hydrogel graft. (**A**) The 2D images of the micro-CT 3D reconstructed femoral condyles following treatment with pbECM hydrogel, DBM, or PBS. Images for each experimental group were chosen to demonstrate the best (**upper panel**) and worst (**lower panel**) bone repair observed from micro-CT scans. Small circles mark the defect area, which is enlarged in the large circles of each image. (**B**) Percentage of the bone volume from the total defect volume of the pbECM hydrogel graft-treated group, DBM graft-treated group, and PBS untreated control group, as derived from the micro-CT images (CTAn Micro-CT Software Version 1.17.7.2). * *p* < 0.05, *** *p* < 0.001.

To address the efficacy of our pbECM hydrogel as a bone graft for treating bone defects, pre-clinical studies were conducted using a femur bone defect rat model. In these studies, a 2.5 mm hole was drilled in the femoral condyle of rats and treated with pbECM hydrogel or PBS as a negative control. As a positive control, commercial DBM was used, which is a well-established clinical standard for bone repair due to its osteoinductive properties. Figure 6A shows the sagittal sectional images of the femoral condyles using micro-CT three weeks after surgery. The red circles mark the defect site. In the upper panel, images were taken from the animal exhibiting the highest level of bone regeneration in each treatment group. The lower panel shows images taken from the animal with the lowest level of bone regeneration in each group, evident from the nearly empty red circles. Notably, animal variability is observed across all groups; however, even within the lowest level of bone regeneration in the pbECM hydrogel group, noticeable bone formation is evident compared to the DBM and PBS groups. To quantify the bone formation within the femur defect site, we calculated the percentage of bone volume from the total volume (% BV/TV) based on the micro-CT images. Figure 6B presents the BV/TV for each animal in each group. In the PBS group, the distribution reflects the natural bone regeneration ability, with approximately one-third of the animals showing almost no new bone formation and only one animal exhibiting 50% BV/TV. The DBM graft group exhibited different levels of new bone formation in the range of 18–55% BV/TV, with only 35% of the animals showing significant regeneration of more than 40% BV/TV. The pbECM-graft group also exhibited variability in bone regeneration among the animals, with 34.5% as the lowest BV/TV value, which is close to the DBM group average (35.5%). Consequently, the bone formation in the pbECM hydrogel treatment group was significantly higher than that of the DBM and PBS groups, which did not significantly differ between them.

#### 2.1.7. Histopathological Assessment of the Bone Defect Regeneration

**Figure 7:**
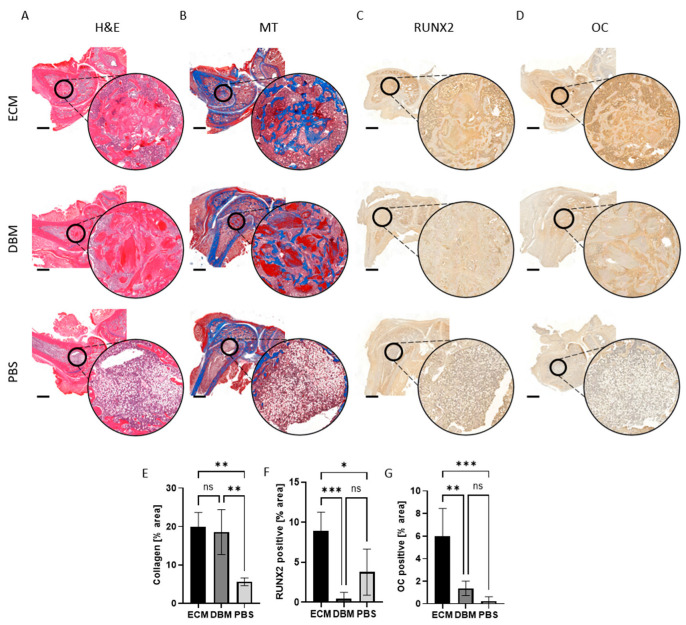
Histopathological assessment of bone defect regeneration. (**A**) H&E and (**B**) Masson’s trichrome histological analysis of the defect site following treatment with pbECM hydrogel, DBM, or PBS. Immunohistochemistry assessment of the regenerated defect site for (**C**) RUNX2 and (**D**) osteocalcin (OC). Scale bars: 2 mm. Small circles mark the defect area, which is enlarged in the large circles of each image. Percentage of (**E**) collagen-stained area, (**F**) RUNX2-stained area, and (**G**) OC-stained area within the femur defect site, quantified from the histological sections (QuPath software V0.5.1). * *p* < 0.05, ** *p* < 0.01, *** *p* < 0.001.

Histopathological staining was conducted to assess the effect of the pbECM hydrogel graft on bone healing, in terms of the migration of cells into the defect area and new bone formation (Figure 7A). H&E staining revealed more pronounced new bone formation in defects treated with the pbECM hydrogel graft compared to those treated with the DBM graft and the PBS untreated control (Figure 7A). In the pbECM graft and the DBM graft groups, both ECM formation and cell migration into the defect were evident, while in the PBS group, only cells were observed. Defects treated with DBM sparsely exhibited fibrous tissue resulting from collagen deposition, which is an early indicator of bone formation. In contrast, the pbECM group exhibited signs of newly formed bone trabeculae structures. Masson’s trichrome staining supported these findings, showing in the defect region a cellular and collagen matrix in both the pbECM and DBM groups, with no significant difference in the collagen’s percentage area between them (Figure 7B,E). Compared to the PBS control, however, both groups exhibited a significantly higher collagen-positive area (Figure 7E).

Immunohistochemistry was utilized to gain a deeper understanding of the obtained regenerative effects and characterize the types of cells that populated the defect area. Primarily, the explants were stained for RUNX2, a transcription factor necessary for initiating the differentiation of MSCs into osteoblasts. The presence of RUNX2 signifies the initiation of new bone formation. Figure 7C revealed that in all groups, cells within the defect site expressed the osteoblast-specific transcription factor RUNX2. However, dramatically higher immunopositivity for RUNX2 was evident in pbECM graft-treated defects compared to the DBM graft group and the PBS group (Figure 7F). Subsequently, the explants were stained for osteocalcin (OC), a marker solely secreted by osteoblasts, the presence of which signifies mature bone formation (Figure 7D). Significantly higher levels of OC-positive cells were observed in the pbECM graft group compared to the DBM and PBS groups, aligning with the micro-CT analysis where bone formation was evident (Figure 7G). Thus, the bone healing effect—demonstrated through multiple evidence of bone formation—was pronounced in the pbECM group compared to the other groups.

### 2.2. Discussion

Various strategies have been explored to develop effective bone grafts, aiming to restore skeletal function in individuals with bone defects. The successful healing of large bone defects requires the presence of osteoprogenitor cells capable of generating new bone, a strong osteoinductive signal to engage and attract these progenitor cells to the injury site, and a supportive matrix that fosters bone formation and encourages angiogenesis [35].

Our suggested approach to meet these requirements involves utilizing the ECM derived from decellularized bones. This approach enables the preservation of the intricate biochemical composition of the natural tissue, which is challenging to recreate using individual building blocks [36]. As the cells of each tissue produce the tissue’s ECM, the ECM inherently upholds the tissue’s balance and functionality [13]. Porcine-derived decellularized bone ECM provides an accessible and standardized source of bone tissue [36], as evidenced by the numerous FDA-approved products based on a porcine-derived decellularized ECM from different tissues [37]. Previous research by our group has also demonstrated the ECM’s versatility and tissue-specific characteristics when employed to fabricate an array of scaffolds, including injectable hydrogels [38], microcapsules [39,40], and patches [41], tailored for diverse biomedical applications. In the current work, we studied the suitability of the pbECM for use as a bone graft and compared it to the hbECM. Various decellularization techniques have been documented for extracting the ECM from different tissues, employing different mechanical, enzymatic, and detergent methods [11,36,40]. In this study, we introduced a unique decellularization protocol that combines demineralization and enzymatic treatment but avoids harsh detergents to minimize the damage to the ECM, preserve its structural and functional integrity, and ensure the retention of essential components, such as collagens and proteoglycans. Our results demonstrate that this combination effectively eliminated visible traces of cellular components, leaving only accepted negligible DNA levels in the isolated bECM from both porcine and human bones [42].

Notably, there was a difference in the initial DNA content between the native porcine and human bone tissues, which can be attributed to the condition of the source bones. While porcine bones are obtained from young, healthy animals that are grown under standardized conditions, human bones are sourced from bone banks and often come from older unhealthy donors. The age and health status of human donors and the processing protocols of the bone bank can significantly affect the overall condition of the bone tissue and its cellularity, potentially leading to a lower initial DNA content. To characterize the resulting pbECM and hbECM, their composition was analyzed. The bECM is known to be composed mainly of collagens (primarily type I), with a minor content of non-collagenous proteins [4], which was also demonstrated in our results of more than 90% collagen content in the pbECM and hbECM. Considering that collagen types I, II, and III are the predominant proteins found in the ECM of various tissues [13,39], it was anticipated that their quantities would be notably high in the bECM of both sources. Indeed, our analysis revealed that collagen I was the most abundant in both the porcine and human bECMs, which aligns with its primary role in maintaining bone strength. However, variations were observed between the porcine and human bECM samples. Collagen types I, II, and III were present in different ratios. Proteoglycans, particularly biglycans, exhibited differential abundance between the two bECMs, with pbECM displaying a higher and more versatile content. These extracellular proteins play pivotal roles in various facets of bone formation, including cell proliferation, osteogenesis, mineral deposition, bone remodeling, and collagen fibrillogenesis [25,43]. Collectively, these proteins contribute to establishing a biologically conducive environment essential for promoting bone regeneration. Similarly to the DNA content, these differences may not necessarily derive from the bone species but could originate from variations in the donors’ age, health status, and the processing methods used for preservation at the bone bank before the decellularization and preparation of the bECM. Using porcine bones, however, these factors can be easily standardized.

When further examining the microstructure of the bECM, it was evident that both bECM samples exhibited a fibrous collagenous arrangement with comparable fiber sizes. The preservation of the fibrous architecture and collagen structure is critical, as it ensures that the mechanical properties and biological functions of the ECM are maintained. These findings underscore the effectiveness of our decellularization protocol in producing bECM scaffolds that closely mimic the native tissue environment. In addition, molecular analysis using FTIR revealed similar amide vibration profiles in both bECM samples, characteristic of collagen-based materials [27]. The analysis further showed that despite the decellularization and processing steps, the structural components essential for the bECM’s function were retained. This structural preservation is vital as it influences the mechanical stability of the scaffold and, most importantly, its interaction with host cells [42]. The fibrous collagen network not only supports cell attachment and proliferation but also plays an important role in guiding new tissue formation, which are key processes in effective bone regeneration [44]. While the decellularization process removed most of the bone minerals, it did not remove all the mineral content. The XRD findings of a low degree of crystallinity in the hbECM and a hydroxyapatite-like spectra in the pbECM can be associated with the age of the bone donors, affecting the composition and properties of bone tissue. With aging, there is a marked increase in bone resorption, leading to a decline in bone mineral density and a significant reduction in bone formation. This is due to the natural aging process, where osteoclast activity outpaces osteoblast activity, resulting in net bone loss [45]. Consequently, the crystallinity of hydroxyapatite tends to decrease with age [46]. This aligns with the lower DNA content in the older tissue source of the hbECM. The residual mineral content, particularly hydroxyapatite, plays an important role in the osteoinductive properties of the bECM. Hydroxyapatite serves as a scaffold for new bone formation and provides essential biochemical signals that promote osteoblast differentiation and bone mineralization [47,48]. The higher crystallinity observed in the pbECM may thus contribute to its superior structural integrity and osteoconductive potential compared to the hbECM, which exhibited a lower degree of crystallinity.

When producing hydrogels from the two bECMs, different rheological behavior was obtained for each hydrogel. Both hydrogels’ behavior indicated the formation of a stable three-dimensional network, essential for providing mechanical support and maintaining the structural integrity of the hydrogel. While both hydrogels displayed typical characteristics of gel-like materials—with the storage modulus consistently exceeding the loss modulus—the pbECM hydrogel demonstrated higher storage and loss moduli compared to the hbECM hydrogel. Hence, the porcine hydrogel forms a stronger and more resilient network, likely due to the higher content of structural proteins such as collagen type I and the higher mineral content. This behavior of both bECM hydrogels aligns with the rheological characteristics of ECM hydrogels derived from various tissues, including heart, artery, pancreas, and urinary bladder [13,40,49]. Furthermore, studies on hydrogels derived from DBM and other biomaterials for bone regeneration have shown similar trends, with storage modulus values often correlating with the degree of crosslinking and protein content [50]. This correlation with the rheological properties of other suggested bone grafts and clinically used materials highlights bECM hydrogels’ potential for bone regeneration [51,52].

Like these different ECM-based hydrogels, the porcine and human bECM-based hydrogels have also supported cell cultivation. The significantly higher proliferation rates on the bECM hydrogels compared to alginate suggest that the native ECM components provide essential biochemical cues that promote cell growth and viability, highlighting their potential as scaffolds for bone tissue engineering.

When implanting a bone graft, the biomaterial can elicit a host response that influences the implant’s integration and biological performance. Therefore, the immunogenic potential of the bECM hydrogels was assessed both in vitro and in vivo. While in vitro neither the pbECM nor the hbECM stimulated macrophages, some differences were seen in the animal studies, with higher cell infiltration and macrophage presence in the hbECM. The implanted pbECM hydrogel showed no signs of eliciting a substantial immune response, as evidenced by the multiple assessed parameters, thus suggesting that its decellularization and processing rendered the pbECM a non-immunogenic biomaterial suitable as a bone xenograft. These results are concordant with prior research by our group and others, which demonstrated the biocompatibility of porcine ECM derived from different tissues [40,41,53,54,55].

The therapeutic potential of our pbECM hydrogel as a bone graft was evaluated in a rat femoral condyle bone defect model, recognized for its clinical relevance in bone regeneration studies, particularly where structural integrity and load-bearing capacity are needed [56]. In addition to the pbECM hydrogel, we included DBM as a comparative standard in our study. DBM is derived from allogeneic bone that has been processed to remove the mineral content while preserving the organic collagen matrix and associated growth factors. DBM has been extensively used in clinical settings due to its proven ability to promote bone healing and its biocompatibility. The osteoinductive properties of DBM make it a benchmark material for evaluating new bone graft substitutes. However, its main drawback is its unpredictable results due to occasional graft resorption [57,58]. By comparing the pbECM hydrogel to DBM, we aimed to assess whether our hydrogel could match or even surpass the osteogenic capabilities of an established bone graft material. In our studies, the superior performance of the pbECM over DBM was evident through the significant enhancement of bone regeneration at the defect site. Rats transplanted with pbECM exhibited substantially higher new bone formation than those implanted with DBM, with all pbECM recipients showing new bone formation of at least 35%, in contrast to only 20% in the DBM group. As expected from the DBM treatment, the results were inconsistent among the animals, ranging from 18% to 55%. Though pbECM treatment did not improve the treatment consistency, the entire range was dramatically improved to 35–82% regenerated bone. Masson’s trichrome staining revealed a substantial presence of collagenous matrix within the defect region in both the pbECM and DBM groups, with comparable percentages of the collagen-positive area between them. This enhanced presence of collagen indicates either robust ECM production or the proper integration of the collagenous grafts and is crucial for structural support and tissue remodeling during bone regeneration processes. The new bone formation observed with pbECM treatment was accompanied by significant cell recruitment, which largely expressed the RUNX2 and osteocalcin markers, signifying MSCs’ differentiation into osteoblasts and differentiated osteoblasts, respectively. This impressive cell recruitment and differentiation is critical to initiating and sustaining proper regenerative processes. Furthermore, it is necessary for graft integration with the host tissue and the remodeling phase of bone healing. Our findings align with previous studies demonstrating the potential of porcine-derived ECM hydrogels for bone regeneration [21,23,24], highlighting their biocompatibility and osteoinductive properties while advancing the field in terms of several key aspects. First, the use of a bone tissue-specific ECM ensures the inclusion of bone-specific proteins and minerals essential for osteogenesis. Second, our comprehensive comparison between porcine and human bone ECM hydrogels reveals that pbECM hydrogels exhibit superior mechanical properties and higher mineral content, both critical for effective bone repair. Finally, our in vivo results demonstrate that pbECM hydrogel significantly enhances bone regeneration compared to commercial DBM, revealing that this hydrogel mimics the natural bone environment and provides relevant biochemical and biophysical cues for cell differentiation and bone formation.

## 3. Conclusions

Our findings highlight the potential of the pbECM hydrogel as a natural bioactive material that preserves the complex composition and molecular structures of the native bone ECM. Derived from a porcine source, this material is highly available, standardized, and consistent while still biocompatible, thus providing a reliable basis for bone grafts. The pbECM hydrogel bone graft not only provided a scaffold for new bone formation but also supported cellular recruitment and differentiation essential for tissue regeneration, thus actively promoting a proper healing process, surpassing existing bone graft materials. Overall, the presented data clearly point to the pbECM hydrogel as a promising bone graft for the treatment of bone defects.

## 4. Materials and Methods

### 4.1. General Reagents

ABC and DAB kits for immunohistochemistry were purchased from Vector Laboratories, Newark, CA, USA. Hydroxyapatite was from Merck (Darmstadt, Germany). Hematoxylin Gill No. 2, hematoxylin Harris, and eosin were purchased from Merck (GH5216, HHS-16, and E6003, respectively). DAPI was from Biotium, Fremont, CA, USA, and Fluoromount-G (DAPI) was from Southern Biotech, Birmingham, AL, USA.

### 4.2. bECM Decellularization

Porcine femur bones were harvested from healthy commercial slaughter-weight pigs (LRI, Lahav, Israel) under the Israeli Animal Welfare (Protection and Experimentation) Law and supervised by the regulatory Israeli National Ethics Committee. Cancellous bones were separated, ground using a bone mill (Smart dentin grinder, KometaBio, Tenafly, NJ, USA), and processed using a modification of our previously reported methods [11,13]. In brief, the bone granules underwent demineralization in 0.5 N hydrochloric acid at room temperature for 24 h. Subsequently, the demineralized bone matrix (DBM) was incubated in a 1:1 mixture of chloroform and methanol (Biolab, Jerusalem, Israel) for 24 h, followed by washes in methanol and distilled water. The DBM was then snap-frozen, lyophilized overnight, and rinsed several times in DDW. To decellularize the lyophilized DBM, it was incubated in a 0.05% trypsin (Merck) and 0.02% ethylenediamine tetraacetic acid (EDTA, Merck) solution at 37 °C for 48 h. The resultant matrix was rinsed in PBS and agitated in a 1% Triton-X-100 (Daejung, Siheung-si, Republic of Korea) solution in 50 mM Tris (Merck) for 48 h. The decellularized bECM underwent freezing in liquid nitrogen followed by lyophilization.

Human femoral heads were collected from healthy individuals as approved by the Helsinki Committee of the Hillel Yaffe Medical Center (0115-20-HYMC). The bones underwent the same procedure as the porcine bones.

### 4.3. DNA Quantification

Residual genetic material (DNA) in the bECM was determined as follows. Whole DNA was extracted using Tri reagent (Merck) and quantified using a PicoGreen kit (Quant-iT PicoGreen dsDNA, Invitrogen, Waltham, MA, USA) according to the manufacturer’s instructions. Fluorescence was measured using an Epoch 2 Microplate Reader (BioTek, Winooski, VT, USA).

### 4.4. Bone-ECM Hydrogel Preparation

To obtain stable bECM hydrogels, the dry bECM was solubilized in HCl (0.01 M) through 1 min sonication and then digested enzymatically using pepsin (1–5 mg mL^−1^, Merck). The solution’s pH was subsequently elevated with NaOH (Biolab, Jerusalem, Israel) and stored at 4 °C till further use. The gelation of the pre-gel was obtained by 1 h incubation at 37 °C. For the generation of alginate hydrogel, an aqueous solution of 2% (*w*/*v*) sodium alginate (MVG, NovaMatrix, Sandvika, Norway) was crosslinked using calcium chloride solution (0.03% *w*/*v*, CaCl_2_, Merck).

### 4.5. Immunofluorescent and Fluorescent Staining of ECM and Cells

Samples were fixed for 20 min in 4% paraformaldehyde (PFA) and then placed in Tissue-Tek^®^ OCT compound (Sakura, Alphen aan den Rijn, the Netherlands), frozen, and sliced for staining (10 μm slices, Leica CM1900 Cryostat, Wetzlar, Germany). Prior to staining, slides were fixed in cold methanol (4 °C) for 20 min. Slides were stained according to the manufacturer’s protocol with primary antibodies: collagen I (1:100, Merck #C2456), collagen IV (1:100, Abcam #ab6586, Cambridge, UK), and collagen V (1:100, Abcam #ab7046). For actin fibers’ staining, samples were stained with phalloidin-TRITC (Sigma-Aldrich, St. Louis, Missouri, USA), and Hoechst 33,258 (Merck) was used for DNA staining. Imaging was performed using the LSM700 confocal microscope (Zeiss, Oberkochen, Germany).

### 4.6. X-Ray Diffraction (XRD)

For XRD analysis, SmartLab 3 kW (Rigaku, Tokyo, Japan) was used with a radiation source of Cu Kα and λ = 1.54 Å (10°–90° range). The results were compared to commercial hydroxyapatite (Merck). The samples were mounted on a holder after being grounded to a fine powder.

### 4.7. Fourier Transform Infrared Spectroscopy (FTIR)

FTIR spectra were captured utilizing a Thermo 6700 FTIR instrument and equipped with a Smart iTR Attenuated Total Reflectance diamond plate at a wave number of 500–3500 cm^−1^ (64 scans at a resolution of 4 cm^−1^). The acquired data were analyzed with OMNIC series software (version 8, Thermo-Scientific, Waltham, MA, USA).

### 4.8. Swelling Test

Lyophilized bECM samples (*n* = 3) were incubated in distilled water at room temperature for 24 h, and the degree of swelling was calculated according to the below formula:

Degree of swelling (%) = (Wet weight − Dry weight)/Dry weight × 100

### 4.9. Thermo-Gravimetric Analysis (TGA)

Lyophilized bECM samples (*n* = 3) were heated at 20 °C/min under a nitrogen atmosphere, from room temperature to 600 °C, using a TGA-Q5500 system (TA Instruments, New Castle, DE, USA). Data were recorded and analyzed using the Trios TA Universal Analysis 200 Software version 4.5A build 4.5.0.5 (TA Instruments, USA).

### 4.10. Scanning Electron Microscopy (SEM)

The bECM was glued to a carbon tape and coated with a thin carbon layer using the Q150TES PLUS carbon evaporation system (Quorum, Laughton, UK). The micrographs were taken using a high-resolution scanning electron microscope Ultra-Plus FEG (Zeiss). The SE2 (secondary electrons) detector was used at an accelerating voltage of 2 kV.

### 4.11. Proteomic Analysis of Porcine and Human ECM

Semi-quantitative proteomic analysis was performed at The Smoler Protein Research Center (Technion—Israel Institute of Technology), as was previously published [13]. Briefly, lyophilized bECM samples were trypsin-digested and analyzed using the Q-Exactive HF mass spectrometer (Thermo-Scientific, MA, USA). The collected data were analyzed using the MaxQuant software 2.1.1.0 using the Andromeda search engine against the sus scrofa and homo sapiens proteomes from the UniProt database.

### 4.12. Mechanical Properties of the bECM Hydrogels

Rheological evaluation of the bECM hydrogels was conducted using a DISCOVERY HR-2 Hybrid Rheometer (TA Instruments). The pre-gel solutions were transferred to the rheometer with a parallel plate geometry (40 mm diameter), and the parameters were set to 1.2 mm gap, 1% strain, 1 rad s^−1^. The temperature was increased to 37 °C to induce gelation, and time sweep analysis was performed for 30 min. The samples were then subjected to frequency sweep analysis ranging from 0.1 to 600 rad s^−1^.

### 4.13. Cell Culture on bECM Hydrogels

bECM pre-gel solutions (100 µL) or alginate (2%) were gelled in a 24-well tissue culture plate and maintained in PBS until seeding. Human bone marrow mesenchymal stem cells (hMSC, Lonza, Basel, Switzerland) of passages 2–6 were cultured in αMEM (biowest, Nuaillé, France), supplemented with 10% fetal bovine serum (FBS, Thermo-Scientific), 1% penicillin/streptomycin, 0.4% amphotericin B (Sartorius, Beit Haemek, Israel), and 0.25 ng/mL basic fibroblast growth factor (PeproTech, Thermo-Scientific). In total, 20,000 hMSC cells/well were seeded, and the cells were cultured for up to 14 days. AlamarBlue™ reagent (AbD Serotec, Kidlington, UK) was used to determine cell viability following the manufacturer’s protocol.

### 4.14. ECM Hydrogel Immunogenicity In Vitro

A macrophage stimulation assay was executed to assess the potential immunogenicity of the bECM and to compare it between human and porcine sources. The RAW macrophage cell line (TIB-71™; ATCC) was cultivated in six-well culture plates (100,000 cells/well) (*n* ≥ 4 wells per group, two independent experiments) in a medium comprising 3 mL of high-glucose DMEM (Sigma) supplemented with 10% FBS, 1% penicillin/streptomycin, and 0.4% amphotericin B (Sartorius). Upon reaching approximately 70% confluence, the culture medium was replaced with a 2% serum medium and incubated overnight. Subsequently, the cells were exposed to 20 mg of pECM, hECM, or PLGA (Sigma-Aldrich). Lipopolysaccharide (LPS, Merck) (1 μg/mL) was used as a positive control, whereas untreated cells were designated as a negative control. After 16 h, the cells’ NO secretion was quantified using the Griess Reagent System (Promega, Madison, WI, USA) following the manufacturer’s protocol. Additionally, real-time RT-PCR was executed to quantify the expression levels of the proinflammatory cytokines TNF-α and IL-1β, utilizing specific primers as follows:

5′-GCCTCCCTCTCATCAGTTCT-3′ and 5′-TGGTGGTTTGCTACGACGTG-3′ for TNF-α. 5′-AGGATGAGGACATGAGCACC-3′ and 5′-ATGGGAACGTCACACACCAG-3′ for IL-1β.

### 4.15. ECM Hydrogel Immunogenicity In Vivo

The immunogenic potential of bECM hydrogels was further assessed in vivo. All animal experiments were approved by the Animal Ethics Committee at the Technion, Israel. The number of animals was determined based on previous experience in our lab. Six-week-old male C57BL mice, acclimated for at least one week, were randomly divided into groups and subcutaneously injected with 100 µL of pbECM hydrogel, hbECM hydrogel, or alginate as a negative control (*n* = 6 mice per group per time point, Envigo, Ness-Ziona, Israel). Mice were sacrificed at 1, 7, and 22 days post-implantation, and cardiac puncture was used to collect blood samples for complete blood count (CBC) and cytokine quantification. CBC was performed by AML lab services, Israel. The remaining blood was centrifuged at 3000 rpm for 10 min, and serum was collected and subjected to quantification of the inflammatory cytokines TNF-α and IL-1β using the mouse cytokine/chemokine magnetic bead panel (Millipore, Burlington, MA, USA). The implanted bECM hydrogels and alginate were collected at scarification for histological evaluation. They were embedded in paraffin blocks, cut to 5 µm thickness sections (Leica RM2255 Microtome, Wetzlar, Germany), and stained using hematoxylin and eosin (H&E), as previously published [59], for pathological evaluation. Immunohistochemical (IHC) staining was performed using F4/80 (Bio-Rad, Hercules, CA, USA) for macrophage and neutrophils presence assessment, respectively.

### 4.16. In Vivo Efficacy Studies

Sixteen-week-old male Wistar rats, acclimated for at least one week, were used (*n* ≥ 6 rats per group in each of the two independent experiments, Envigo, Israel). The number of animals per group was determined based on a power analysis with 1-β = 0.8, α = 0.5. Under general isoflurane anesthesia, the femoral condyle of each rat was exposed, and a 2.5 mm hole was drilled in the bone. In two animals, the bone was injured by the drilling, and they were excluded from the experiment. The drilled defects were filled with pbECM hydrogel, commercial DBM (AlloFuse DBM, AlloSource, Centennial, CO, USA), or PBS as control, and the fascia and skin were then closed. Allocation into treatment groups was random. After 3 weeks, rats were sacrificed and all femurs with bone defects were dissected and soaked in neutral buffered formalin (NBF, 10%, Sigma) for 24 h. Following this, the femurs were subjected to micro-computed tomography (CT) and histological and histochemical examinations as follows.

Micro-CT images of the bone defects were obtained using a micro-CT scanner (voltage: 70 kV, current: 70 μA, pixel size: 17 μm/pixel, Bruker SkyScan 1276, Billerica, MA, USA) and reconstructed using the NRecon software interface (Bruker, version 1.7.3.1). Blinded quantification analysis of the percentage of bone volume out of the total defect volume (BV/TV) was achieved using image analysis with CTAn Micro-CT Software (Bruker, version 1.17.7.2). A total of 5 animals were excluded from the analysis: 2 were found to have broken bones upon CT evaluation, and 3 were excluded due to incorrect drilling positions observed in the CT scans.

At the end of the studies, femora were harvested and decalcified with 20% EDTA solution at 4 °C and dehydrated with a graded series of ethanol treatments before being embedded in paraffin. Paraffin sections of 5 µm thickness were cut (Leica RM2255 Microtome, Wetzlar, Germany), stained with H&E or Masson’s trichrome, and scanned using a slide scanner (Pannoramic 250 Flash II, 3DHistech, Budapest, Hungary).

### 4.17. Immunohistochemistry Analyses

Immunohistochemical (IHC) staining was performed using RUNX2 and OC primary antibodies (Santa Cruz Biotechnology, Dallas, TX, USA). Samples were then stained using a biotinylated secondary antibody goat anti-mouse (Vector). Finally, samples were counterstained using Gil’s hematoxylin for nuclei detection. All slides were then scanned using the slide scanner (Pannoramic 250 Flash II, 3DHistech, Hungary). For the quantification of RUNX2 and OC, QuPath version 0.5.0 was used [60]. The algorithm for positive DAB detection was based on a pixel classifier and trained on representative pictures with dedicated annotations. The quantification was performed in a defined region of interest (ROI) (area = 4,910,208.258 µm^2^). Intensity thresholds for pixel detection and classification were manually set and performed identically for all samples of each staining type. Pixel densities were estimated as the percentage of positive pixels per µm^2^ of surface area.

### 4.18. Statistical Analysis

For every experimental group and time point, the data are shown as the mean ± standard deviation from at least three repetitions, unless otherwise indicated. A *t*-test was used to determine the statistical difference between means for individual comparisons, and a two-way ANOVA was used for multiple comparisons, utilizing the Holm–Sidak method or Tukey’s test, respectively. Unless otherwise noted, *p*-values < 0.05 were considered significant. In every experiment, representative micrographs were chosen from all samples.

## Figures and Tables

**Table 1:** Proinflammatory cytokines. Serum levels of the proinflammatory cytokines TNF-α, IL-1β, IFN-γ, and IL-6 in all experimental groups. * *p* < 0.05, ** *p* < 0.01.

	Treatment	TNF-α	IL-1β	IFN-γ	IL-6
24 h	pECM	17.3 ± 2.2	20.8 ± 3.0	8.8 ± 3.6	12.7 ± 5.4
	hECM	14.6 ± 2.0	15.7 ± 1.4 *	6.5 ± 2.0	13.4 ± 1.4 **
	Alginate	16.9 ± 3.5	21.9 ± 5.0 *	14.2 ± 8.4	21.0 ± 1.2 **
7 days	pECM	14.5 ± 2.6	14.9 ± 3.3	20.3 ± 7.5	15.2 ± 2.7 **
	hECM	15.0 ± 0.4	17.6 ± 0.5	17.4 ± 4.8	13.6 ± 5.7
	Alginate	14.1 ± 3.7	16.8 ± 2.0	17.9 ± 4.5	7.4 ± 1.2 **
22 days	pECM	13.0 ± 1.4 *	15.5 ± 2.8 *	12.1 ± 7.7	8.7 ± 1.9
	hECM	6.6 ± 1.9 *	9.7 ± 0.8 *	5.5 ± 0.0	6.1 ± 1.9
	Alginate	9.9 ± 3.0	13.3 ± 4.3	5.5 ± 0.0	8.1 ± 3.1

## Data Availability

The original contributions presented in this study are included in the article/Supplementary Materials. Further inquiries can be directed to the corresponding author.

## Supplementary Materials

The following supporting information can be downloaded at: https://www.mdpi.com/article/10.3390/gels11030173/s1, Figure S1: Swelling degree; Figure S2: Thermo-gravimetric analysis.
