# Supplementary material for: Porcine Bone Extracellular Matrix Hydrogel as a Promising Graft for Bone Regeneration
Source: Gels. 2025 Feb 27;11(3):173. doi: 10.3390/gels11030173 (PMC11942433; doi:10.3390/gels11030173)
Supplement: Supplementary file 1 [file gels-11-00173-s001.zip › gels-3463181-supplementary.pdf]

Porcine Bone ECM Hydrogel as a Promising Graft for Bone Regeneration

*Rotem Hayam, Shani Hamias, Michal Skitel Moshe, Tzila Davidov, Feng-Chun Yen, Limor Baruch, and Marcelle Machluf**

Supplementary Material

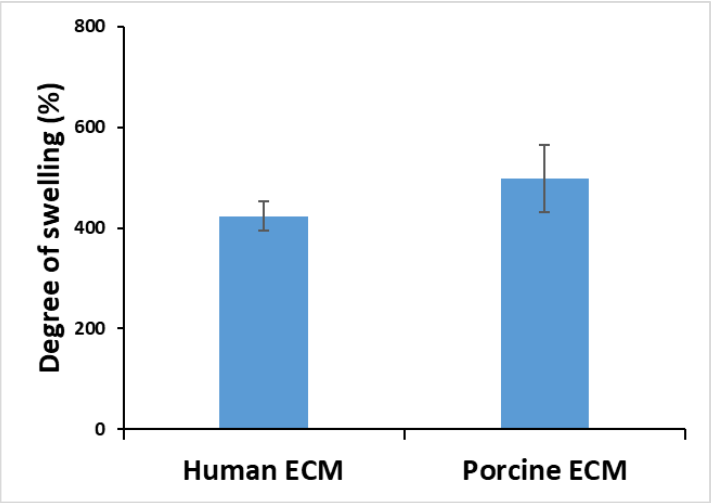

Supplementary Figure S1: Swelling degree. The degree of swelling of lyophilized human and porcine bone ECM (n=3).

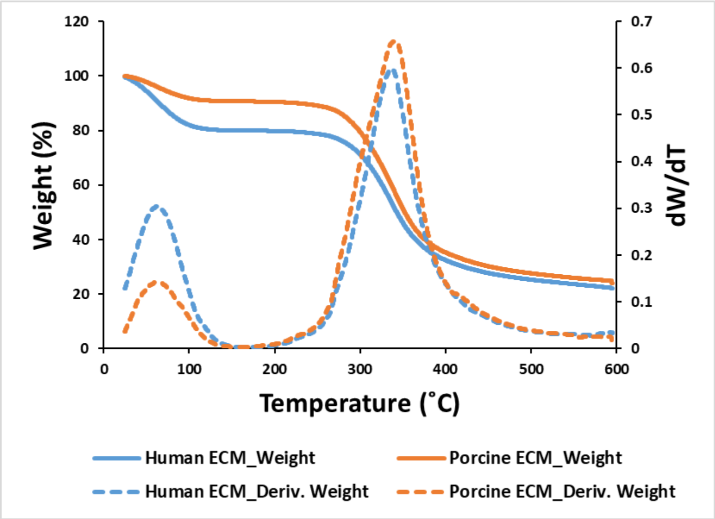

Supplementary Figure S2: Thermo-gravimetric analysis. TGA thermograms of lyophilized human and porcine bone ECM and their first derivatives (n=3).
